# Intravenous Application of a Primary Sevoflurane Metabolite Improves Outcome in Murine Septic Peritonitis: First Results

**DOI:** 10.1371/journal.pone.0072057

**Published:** 2013-08-19

**Authors:** Inge K. Herrmann, Maricela Castellon, David E. Schwartz, Melanie Hasler, Martin Urner, Guochang Hu, Richard D. Minshall, Beatrice Beck-Schimmer

**Affiliations:** 1 Institute of Anesthesiology, University Hospital Zurich, Zurich, Switzerland; 2 Institute of Physiology and Zurich Center for Integrative Human Physiology, University of Zurich, Zurich, Switzerland; 3 Department of Anesthesiology, University of Illinois College of Medicine at Chicago, Chicago, Illinois, United States of America; 4 Department of Pharmacology, University of Illinois at Chicago, Chicago, Illinois, United States of America; University of Cincinnati, United States of America

## Abstract

Volatile anesthetics are known to have immunomodulatory effects in conditions of organ injury. A recent study in an experimental sepsis model has shown remarkably improved survival when mice were exposed to volatile anesthetics. In the present study, we show that hexafluoroisopropanol – a water-soluble primary sevoflurane metabolite – has beneficial effects on the overall survival in a murine model of cecal ligation and puncture. Seven-day survival as well as tissue damage markers including transaminases and high mobility group box protein-1 were assessed as measures of end organ damage. In animals undergoing cecal ligation and puncture procedure hexafluoroisopropanol conditioning - but not late postconditioning 24 hours after sepsis induction - significantly increased survival rate (17% vs. 77%, p = 0.037) and attenuated secretion of organ damage markers. This study shows survival benefits by administration of the metabolite of a volatile anesthetic. If successfully translated, hexafluoroisopropanol might offer interesting therapeutic opportunities in the future treatment of abdominal sepsis.

## Introduction

Volatile anesthetics have been shown to positively affect outcome after ischemia reperfusion injuries in both clinical and basic research studies [1], [2], [3]. Most recently, it has been demonstrated in an experimental sepsis model that conditioning with desflurane or sevoflurane and post-conditioning with sevoflurane improves survival in murine septic peritonitis [4]. We now found similar effects by intravenous administration of hexafluoroisopropanol (HFIP, water-soluble trifluorinated small molecule, (CF_3_)_2_CHOH), a primary metabolite of sevoflurane, instead. Trifluorinated small molecules have previously been shown to exert immunomodulatory effects similar to volatile anesthetics in an *in vitro* model of lipopolysaccharide (LPS)-induced cell injury [5]. However, only successful translation into an *in vivo* model allows first evaluation of the significance of these results. In the present study, we investigated in a state-of-the-art model of murine sepsis with intra-abdominal focus (peritonitis) whether HFIP application improves overall survival of septic animals. Tissue damage markers including blood urea, transaminases and high mobility group box protein-1 (HMGB-1) were determined as measures for end organ damage.

## Materials and Methods

### Ethics Statement

Animal experiments were approved by the University of Illinois Animal Care and Use Committee (Chicago, IL, USA). Experiments were performed following the guidelines from the Association for Assessment and Accreditation of Laboratory Animal Care. C57BL/6 mice were closely monitored before and during the experiments. Mice received buprenorphine (0.1 mg/kg subcutaneously) immediately after surgery and as needed thereafter for analgesia. Severely moribund animals were euthanized.

### Animals

Eight to 12 week old male C57BL/6 mice (Charles River Laboratories, Chicago, IL, USA) were used for experiments.

### Anesthesia and Sepsis Induction by Cecal Ligation and Puncture

Cecal ligation and puncture (CLP) was carried out under ketamine/xylazine anesthesia as described previously [4]. Briefly, the distal 20% (below the ileocecal valve, about 1 cm from the tip) of the cecum was ligated with a 6-0 suture. The cecum was punctured through and through four times with a 20G needle. The experiments were part of a series and the same CLP group was used as in recent work due to ethical considerations [4]. In CLP animals, saline was infused at 20 mL/kg/h (N = 12). For HFIP conditioning (N = 12), saline (20 mL/kg/h) containing HFIP was administered through the right external jugular vein for 30 min, immediately after CLP induction under ketamine/xylazine anesthesia (0.015 or 0.04 mg HFIP/g body weight). The animals were closely monitored and the depth and duration of anesthesia was comparable for all groups. HFIP doses were based on *in vitro* results and further reduced after observing lethal effects in (severely compromised) CLP animals. The dose of 0.015 mg/g is well below the reported LD_50_ value in (healthy) mice (0.18 mg HFIP intravenous per g BW as reported by U.S. Army Armament Research & Development Command, Chemical Systems Laboratory, NIOSH Exchange Chemicals. Vol. NX#03623).

For postconditioning experiments 24 hours after CLP induction, mice were anesthetized using ketamine (100 mg/kg, no xylazine) for the intravenous administration of HFIP. In the 0.015 mg HFIP/g group 100% mortality was observed within 48 hrs (N = 8). Therefore, similarly to the sevoflurane experiments in the previous study [4], a reduced dose of 0.0075 mg HFIP/g was administered at the 24 hours time point.

### Survival Study

For survival studies, animals were observed for up to 7 days at <8 hrs intervals.

### Blood Analysis 24 hours after Sepsis Induction

In additional experiments mice were harvested 24 hours after CLP-induction (N = 6 per group). Blood samples were analyzed to assess organ damage markers (Hitachi 916 chemistry analyzer, Roche Diagnostics, Laval, Quebec, Canada). Inflammatory mediators such as interleukin-6 (IL-6), monocyte chemoattractant protein-1 (MCP-1) (both from BD Biosciences San Diego, CA) as well as HMGB-1 (IBL International; Hamburg, Germany) were measured following the manufacturer’s protocol.

### Statistical Analysis

Survival data was analyzed using Bonferroni corrected log rank tests in Origin (Kaplan Meier Survival Analysis, OriginLAB, Northampton, MA, USA). Markers for organ function for treated and untreated groups were compared using Mann-Whitney U tests (two-tailed) and Bonferroni correction for multiple comparisons. Because of heteroscedasticity, rank transformation was performed before the inflammatory mediator data set was analyzed using linear regression analysis (inflammatory mediator expression as dependent variable, affiliations to the respective treatment group were coded as independent, dummy-coded variables). P-values ≤0.05 were considered statistically significant.

## Results and Discussion

**Figure 1:**
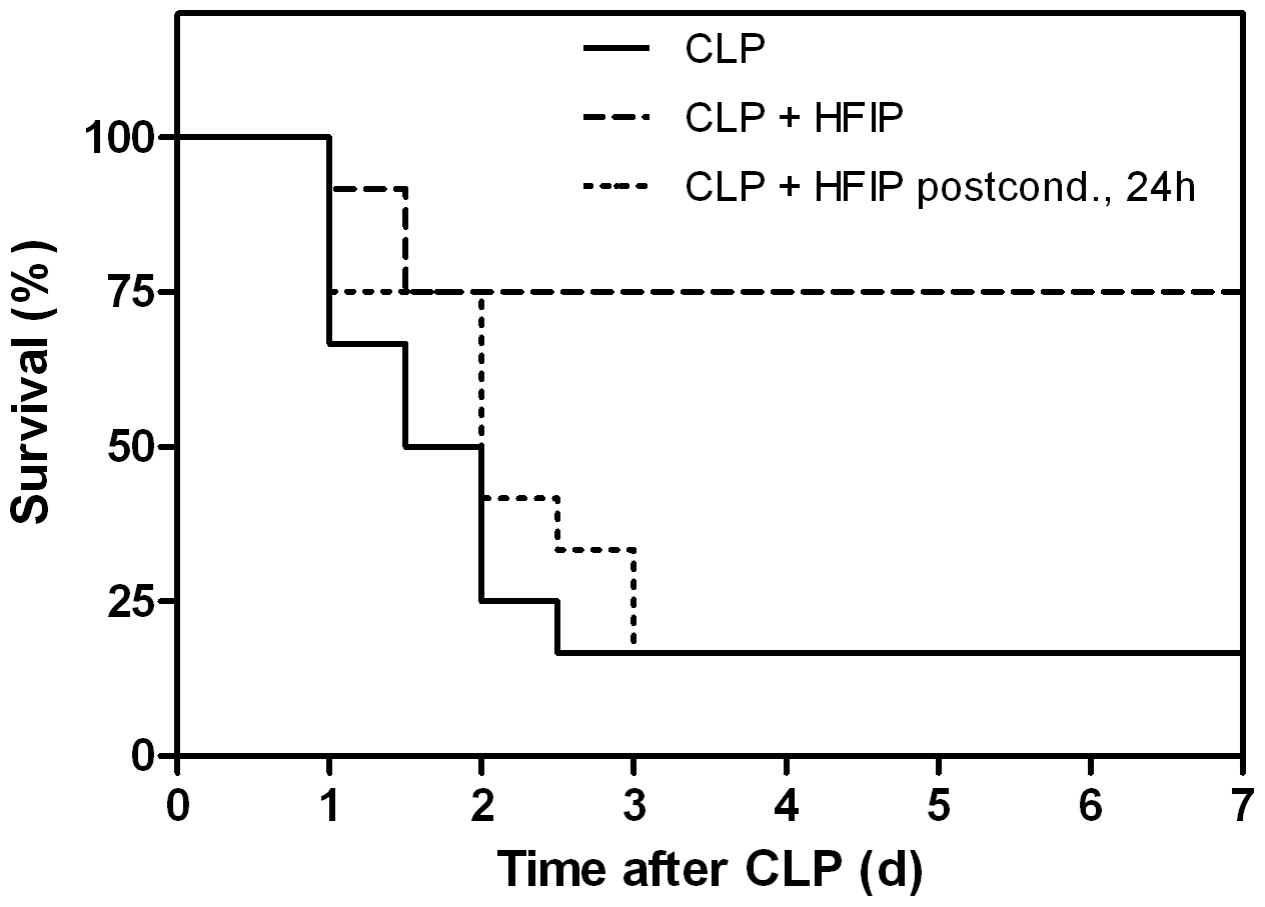
Survival analysis. Seven day survival of CLP mice, CLP mice receiving HFIP immediately after CLP induction (CLP+HFIP, N = 12) (CLP vs. CLP+HFIP, p = 0.037) and CLP animals receiving HFIP 24 hours after sepsis induction (CLP+HFIP postcond., 24 h, N = 12) (CLP vs. CLP+HFIP postcond., 24 h, ns) CLP: cecal ligation and puncture. HFIP: hexafluoroisopropanol. ns: non –significant.

**Figure 2:**
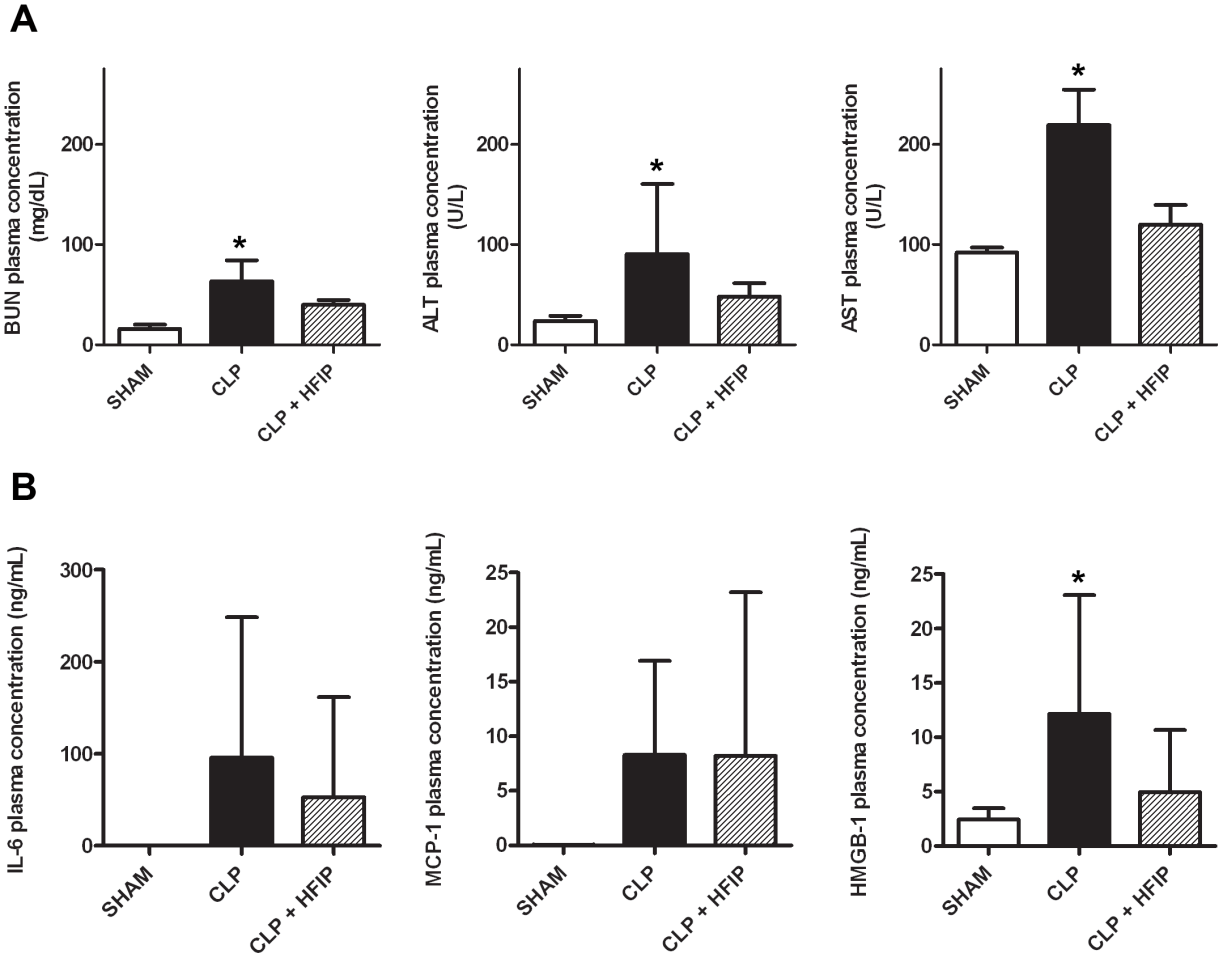
Organ damage and Inflammation. Plasma levels of organ damage markers (blood urea nitrogen (BUN), alanine aminotransferase (ALT) and aspartate aminotransferase (AST)) (**A**) and inflammatory mediators (interleukin-6 (IL-6), monocyte chemoattractant protein-1 (MCP-1) and high mobility group box protein-1 (HMGB-1)) (**B**) for the experimental groups (N = 6) 24 hours after sepsis induction. CLP: cecal ligation and puncture. HFIP: hexafluoroisopropanol. IL-6: * denotes p<0.05 compared to SHAM.

In a murine CLP sepsis model we found that intravenous administration of water-soluble HFIP showed markedly improved overall 7-day survival (77%) compared to the CLP group (17%) when administered in saline at a dose of 0.015 mg/g immediately after CLP surgery (p = 0.037, N = 12) (**Figure 1**). SHAM-operated mice tolerated HFIP doses of at least 0.08 mg/g. However, in animals undergoing CLP procedure the administration of doses ≥0.04 mg/g HFIP was associated with a mortality of 100% by day 2. The measurement of biochemical parameters in plasma 24 hours after CLP-induction revealed that HFIP administration significantly attenuated CLP-induced organ injury (**Figure 2**). Increased levels of blood urea nitrogen (BUN, p = 0.010), alanine aminotransferase (ALT, p = 0.037) and aspartate aminotransferase (AST, p = 0.001) but not of alkaline phosphatase (ALP), were found in the CLP group compared to SHAM animals 24 hours after induction of sepsis. In CLP+HFIP group, mean ALT, AST and BUN levels were comparable to the SHAM group, suggesting that HFIP significantly attenuated CLP-induced organ injury. Plasma HMGB-1, representing a late phase proinflammatory mediator in the pathogenesis of sepsis [6], [7] was upregulated fivefold in the CLP group at the 24 hours time point compared to SHAM animals (p = 0.005, R^2^ = 0.349). In CLP mice that received HFIP plasma HMGB-1 levels were significantly lower and not different from those in SHAM animals (p = 0.81, R^2^ = 0.349). No significant differences were found neither in plasma levels of early inflammatory mediators (IL-6 and MCP-1) nor in bacterial counts among the different groups. When HFIP was administered 24 hours after sepsis induction (late post-conditioning), no statistically significant differences in overall survival were observed for both doses (0.015 mg/g and 0.0075 mg/g). At the 24 hours time point, mice showed pronounced symptoms of sepsis and the ketamine anesthesia required for catheter placement might have additionally compromised the outcome in the late postconditioning group.

In summary, intravenous administration of the primary sevoflurane metabolite HFIP provides significant protection against severe septic peritonitis in a murine model. HFIP not only decreases levels of surrogate markers, but also improves outcome in the present animal model of experimental sepsis.

Underlying mechanisms of protection in septic peritonitis remain to be investigated for both volatile anesthetics and HFIP. HMGB-1 has recently been identified as a systemic marker with prognostic impact that is released by necrotic and severely stressed cells and functions as a late inflammatory mediator peaking considerably later than e.g. IL-6 or tumor necrosis factor (TNFα) [7], [8] and the observed protection is possibly mediated through attenuated secretion of HMGB-1. This in good agreement with previous studies which found significant protection against lethal endotoxemia, sepsis, and LPS-induced acute lung injury, with rodents being protected from development of organ damage and failure through decreased extracellular levels of (free) HMGB-1 achieved by e.g. passive immunization, even when the HMGB-1 antibody administration was delayed [8], [9]. In contrast to the antagonization of early cytokines such as TNFα, therapeutic neutralization of HMGB-1 has shown encouraging results in experimental models of arthritis and sepsis, and may thus be a key determinant for the rescue of septic animals [8], [10].

### Conclusion

In conclusion, the present study shows beneficial effects on survival using a primary sevoflurane metabolite, HFIP, in a sepsis model currently considered as the gold standard in experimental sepsis. This is the first experimental study demonstrating a survival benefit by administration of a volatile anesthetics metabolite. In contrast to volatile anesthetics the metabolite is water-soluble and intravenously injectable, which greatly extends the spectrum of possible future applications to non-theater environments [4]. As the intravascular application of volatile anesthetics is potentially lethal and requires complex formulation [11], structure-similar water-soluble compounds have the potential to overcome these limitations. Validation in other animal models and insight into the mechanism of action is now required before the actual therapeutic relevance can be appreciated.
